# Real-World Outcomes of Inhaled Treprostinil in Pulmonary Hypertension Related to Interstitial Lung Disease: A Multicenter, Retrospective Analysis

**DOI:** 10.3390/jcdd13030129

**Published:** 2026-03-10

**Authors:** Andrew R. Kyle, Arun Jose, Kristen Catherman, Jean Elwing, Roxana Sulica, Gerald S. Zavorsky, Namita Sood

**Affiliations:** 1 Division of Pulmonary, Critical Care, and Sleep Medicine, University of California-Davis, Sacramento, CA 95817, USA; nsood@health.ucdavis.edu; 2 Division of Pulmonary and Critical Care Medicine, University of Cincinnati, Cincinnati, OH 45219, USA; 3 Division of Pulmonary and Critical Care Medicine, New York University Langone Health, New York, NY 10016, USA; 4 Department of Physiology and Membrane Biology, University of California-Davis, Davis, CA 95616, USA

**Keywords:** pulmonary hypertension, interstitial lung disease, inhaled treprostinil

## Abstract

Inhaled Treprostinil is the primary treatment of pulmonary hypertension related to interstitial lung disease (PH-ILD). Despite treatment effectiveness in clinical trials, the real-world safety and tolerability of this therapy remains unclear. We conducted a multicenter, retrospective review of adults with PH-ILD who were prescribed inhaled treprostinil. We assessed clinical outcomes, 6 min walk distance (6MWD) and changes in natriuretic peptides (BNP, NT-proBNP), as well as medication tolerance. Eighty-three patients met the inclusion criteria. The 6MWD data was collected but a limited number of patients had results within close proximity to initiation of inhalational treprostinil with only seven patients having assessments within the 3 months prior to initiation as well as 3 months post therapy. Limited 6MWD data is likely due, in part, to coinciding with the COVID pandemic, limiting face-to-face interactions and exercise testing. The majority of our subjects, 63%, had an absolute improvement in their BNP level, over a mean duration of 170 days. However, no significant difference was detected between baseline and follow-up natriuretic peptide levels. Adherence was assessed and the majority (77%) of patients remained on therapy at the time of censoring, with three-quarters (75%) meeting the target dose. Of the 15 patients intolerant to nebulized treprostinil who were transitioned to a dry powder inhaler, the majority (87%) were able to tolerate the other formulation. The medication was well-tolerated with a large percentage of patients remaining on therapy indefinitely and reaching the targeted therapeutic dose.

## 1. Introduction

Pulmonary hypertension (PH), characterized by elevated pulmonary arterial pressures, is a common complication of interstitial lung disease (ILD) and is associated with increased morbidity and mortality [1,2,3]. Pulmonary hypertension associated with ILD (PH-ILD) represents a subset of WHO Group 3 pulmonary hypertension characterized by precapillary PH complicating various forms of chronic parenchymal lung disease and/or hypoxemia [1,2]. The 2022 European guidelines notably changed the terminology from “due to” to “associated with” lung disease, reflecting growing recognition that pulmonary vascular disease can develop and progress independently of the underlying parenchymal process [3]. PH-ILD can complicate multiple forms of interstitial lung disease, including idiopathic pulmonary fibrosis, chronic hypersensitivity pneumonitis, nonspecific interstitial pneumonia, combined pulmonary fibrosis and emphysema, and connective tissue disease-associated ILD, with a prevalence up to 40–60% in various patient populations with ILD [4,5,6,7,8]. Among all WHO groups of pulmonary hypertension, PH-ILD carries the worst prognosis, with development of PH associated with increased supplemental oxygen requirements, reduced exercise capacity, decreased quality of life, and earlier death compared to ILD alone [6,7,8,9]. The pathophysiology of PH-ILD differs fundamentally from pulmonary arterial hypertension (PAH), WHO Group 1 PH. The underlying pulmonary vasculopathy involves loss of the small pulmonary vascular bed, chronic alveolar hypoxemia, and pulmonary vascular remodeling [2,9]. In some patients, pulmonary vascular disease dominates the clinical presentation—a phenomenon termed the “pulmonary vascular phenotype” [2]. These patients may exhibit disproportionately severe pulmonary hypertension relative to the degree of parenchymal lung disease, with hemodynamic impairment that cannot be fully explained by hypoxia or lung function abnormalities alone [1,2,4,9]. Diagnosis of PH-ILD requires a high index of suspicion given substantial symptom overlap between ILD and PH. In patients with ILD, development of PH is suggested by reduced diffusing lung capacity (DLCO), impaired 6 min walk distance (6MWD), pronounced exertional desaturation, and impaired heart rate recovery after exercise [1,2,4,10]. While transthoracic echocardiography serves as the most common screening tool, it lacks sensitivity and specificity [4]. Right heart catheterization remains the gold standard for confirming precapillary PH-ILD, demonstrating elevated mean pulmonary arterial pressure (≥25 mm Hg historically, though recent definitions use ≥20 mm Hg), elevated pulmonary vascular resistance (>2 Wood units, historically > 3 Wood units), and normal pulmonary artery wedge pressure (≤15 mm Hg) [1,2,10]. Historically, severe PH-ILD was defined as mean pulmonary artery pressure (mPAP) of ≥35 mmHg, or mPAP ≥ 25 mmHg with a low cardiac index (<2.5 L/min/m^2^); however, it is currently defined as a precapillary disease with PVR > 5 Wood units [3,4].

The mainstay of treatment for PH-ILD centers around inhalation of the pulmonary vasodilator treprostinil, which has been shown in the landmark INCREASE trial to improve functional capacity (6MWD), ameliorate PH disease severity, and reduce the risk of clinical worsening [11]. Based on INCREASE trial findings, inhaled treprostinil has been approved for use in PH-ILD. The drug is currently available in three formulations; treprostinil nebulizer (Tyvaso), treprostinil dry powder inhaler (Tyvaso DPI), and a dry inhalation powder formulation (Yutrepia), where treprostinil is designed using proprietary technology (PRINT), which aims to produce drug particles that are uniform in size, shape, and composition [12]. Another formulation of inhaled treprostinil powder, treprostinil palmitil, is currently in advanced stages of drug development [12]. Despite these clinical benefits demonstrated in the INCREASE trial, treatment with inhaled treprostinil is associated with side-effects including cough and dyspnea; titration to doses at which therapeutic benefit can be realized may not always be feasible outside of a clinical trial [13]. To address this gap in knowledge, we conducted a multi-center, retrospective study assessing the real-world efficacy, safety and tolerability of inhaled treprostinil therapy in patients with PH-ILD. Formulations used in this study were treprostinil nebulizer (Tyvaso) and treprostinil dry powder inhaler (Tyvaso DPI), since Yutrepia had not been approved at the time of data collection.

## 2. Materials and Methods

This was a multicenter, retrospective study of patients with PH-ILD on inhaled treprostinil at three participating university centers. Approval of the study was granted by the Institutional Review Board (IRB) of all three sites separately (IRB: 2013583-2, IRB 2020-1103, IRB: 023-00947).

Eligible participants were adults aged 18 years or older, with a clinical diagnosis of both PH and ILD, prescribed inhaled treprostinil by a pulmonary hypertension specialist, between 1 January 2020, and 1 February 2024. The type and severity of interstitial lung disease (ILD) were assessed using pulmonary function tests (PFTs), high-resolution chest computed tomography (HRCT), and relevant clinical and/or histopathological data, as determined by the judgment of the referring pulmonologist. PH was diagnosed by right heart catheterization. Patients were eligible for inclusion if they had precapillary PH, defined at the time of study enrollment as a mean pulmonary artery pressure (mPAP) ≥ 25 mm Hg, pulmonary vascular resistance (PVR) > 3 Wood units, and pulmonary artery wedge pressure ≤ 15 mm Hg, in accordance with contemporaneous U.S. regulatory approval criteria. Integration of data from ancillary investigations (e.g., echocardiography, HRCT, and PFTs), diagnostic classification of PH-ILD, and treatment decisions were performed by the PH Specialty Team at the enrolling center. Complex clinical cases, where indicated, were reviewed in multidisciplinary conferences involving PH specialists, pulmonologists, cardiologists, radiologists, pathologists, and rheumatologists as appropriate. Participants were excluded if previously enrolled in the INCREASE randomized controlled study.

Clinical data were extracted from the electronic medical record including demographics (age, sex, etiology of ILD, and comorbidities), baseline disease severity (right heart catheterization hemodynamics, echocardiogram and PFTs values, HRCT findings, laboratory values (natriuretic peptides), and functional capacity (6MWD). Specifics on inhaled treprostinil therapy (delivery method, peak dose, side effects, and tolerability) and clinical outcomes (mortality, transplantation, cardiopulmonary hospitalization, and discontinuation of inhaled treprostinil therapy) were also obtained from the medical record. Concomitant anti-fibrotic therapy (pirfenidone or nintedanib) was also captured. Baseline data values were the closest to the initiation of inhaled treprostinil therapy.

Although this real-world study did not include a prespecified primary endpoint, the primary outcome of interest was real-world effectiveness of inhaled treprostinil in PH-ILD. Secondary outcomes included treatment tolerance, side effects, and persistence on therapy. Clinical outcomes of interest included changes in 6MWD and BNP and NT-proBNP following therapy initiation. Although 6MWD was assessed, heterogeneity in the timing of assessments limited interpretability. Patients were included in natriuretic peptide outcome analyses only if both baseline and follow-up BNP or NT-proBNP values were obtained within twelve months of treatment initiation. Tolerance of the drug—in both nebulized and dry power formulation—maximal dose achieved, side effect profile, reasons for discontinuation, and persistence on the drug were analyzed throughout the duration of drug exposure.

### Statistical Analyses

To evaluate changes in 6MWD, BNP, and NT-proBNP values between baseline and follow-up, we used linear mixed-effects models. The primary outcome variables were 6MWD, BNP, and NT-proBNP, with timepoint (baseline vs. follow-up) serving as the fixed effect. Random intercepts were included for both subject (to represent individual patients) and site (to account for the three hospital sites), allowing the model to adjust for patient-level and site-specific variability.

Models were fit using restricted maximum likelihood (REML), with the significance of the fixed effects assessed via *t*-tests using Satterthwaite’s approximation for degrees of freedom. In addition, 95% confidence intervals for the fixed effects were generated through parametric bootstrapping with 1000 samples, providing estimates of uncertainty around effect sizes.

To assess for potential bias, given the reduced number of patients that had outcome data, we compared baseline characteristics between patients with and without follow-up measurements.

Due to the wide variability in follow-up intervals, we conducted sensitivity analyses to include follow-up duration as a covariate and stratified by follow-up duration.

Statistical analyses were conducted using RStudio 2024.04.2 Build 764 (2 April 2024 Build 764) using tidyr, dplyr, lme4, lmerTest, and boot packages. Figures were generated using GraphPad Prism (version 10.3.1.509 for Windows, GraphPad Software, Boston, MA, USA). A two-sided *p*-value of <0.05 was considered statistically significant.

## 3. Results

Eighty-three patients met the inclusion criteria. Baseline demographic data and disease severity are reported in Table 1. The median age of the cohort was 73 years old, there was a slight predominance of male subjects (54.2%), and the majority of the cohort self-identified as White (73.5%). The most common etiology of lung disease was idiopathic pulmonary fibrosis (34%) followed by connective tissue disease (22%). Thirty-four percent of patients were on an antifibrotic and 35% received immunosuppression.

Hemodynamic severity of PH was moderate to severe when considering both definitions, with an average mean pulmonary artery pressure of 34 mmHg, CI (cardiac index) of 2.5 L/min/m^2^, and PVR of 5.3 Wood units. The majority of the cohort (N = 75, 90%) was initially started on the inhaled nebulizer, iNeb, formulation of treprostinil (Table 2).

### 3.1. Change in 6 min Walk Distance

Out of the 83 patients included, 48 patients had 6MWD measurement before and after initiation on inhaled Treprostinil. Pre-treprostinil walk distances ranged from 150 to 537 m and post-treprostinil walk distances ranged from 90 to 506 m. Pre-treatment walk testing occurred between 0 and 1356 days prior to therapy initiation, while post-treatment walk testing occurred between 1 and 1080 days after initiation, highlighting substantial heterogeneity in the functional status and timing of the assessment. A total of 37 patients had both baseline and follow-up 6MWD measurements obtained within twelve months of starting therapy and were included in descriptive analyses. The median interval between baseline and follow-up 6MWD assessments was 203 days (interquartile range [IQR], 138–252 days), and the median time from baseline 6MWD assessment to treprostinil initiation was 64 days (IQR, 27–108 days). Overall, the median baseline 6MWD was 270 m (IQR, 146–318 m).

Only seven patients had 6MWD assessments obtained within three months both before and after treatment initiation, and only one patient had both baseline and follow-up 6MWD assessments performed within one month before and after starting therapy. Due to the limited number of patients with tightly timed assessments and the heterogeneity in testing intervals, formal longitudinal analyses of 6MWD were limited, and results should be interpreted cautiously; additional analyses were not undertaken.

### 3.2. Change in Natriuretic Peptides

**Figure 1:**
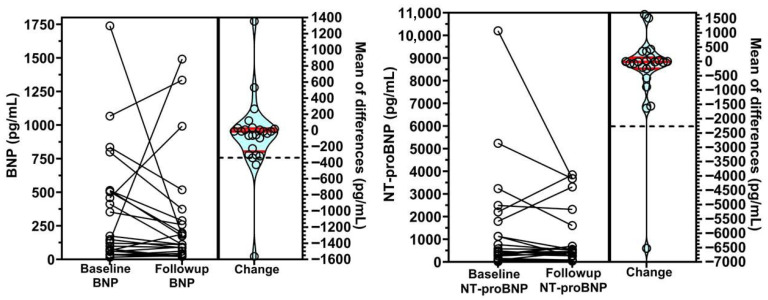
Changes in BNP and NT-proBNP levels from baseline to follow-up. (**Left**) Panel: The left panel displays the changes in B-type natriuretic peptide (BNP) levels between baseline and follow-up assessments. Individual patient data points are connected by lines to illustrate the change in BNP for each subject. The (**right**) section of the panel shows a violin plot representing the distribution of the changes in BNP levels. The dashed black line indicates the mean change, while the solid red lines represent the upper quartile, median, and lower quartile of the distribution, respectively. The change in BNP levels from baseline to follow-up was not statistically significant (Estimate = −12 pg/mL, 95% CI = −224 to +8 m, *p* = 0.536), indicating no significant difference between baseline and follow-up BNP levels.

A subset of 25 patients had BNP levels recorded within the prespecified timeframe. Median duration between tests was 165 days (IQR, 79–244). The median baseline BNP of the subset was 137 pg/mL (IQR 58–503), which on average decreased over time by 12 pg/mL (95% CI = −224 to 8 pg/mL, *p* = 0.536), though this trend failed to reach significance. In the majority of subjects in this subset (N = 16, 64%), however, BNP did decrease over time (Figure 1).

A subset of 23 subjects had available NT-proBNP data meeting timeline restrictions. Median duration between tests was 101 days (IQR, 82–213). Baseline median NT-proBNP of this subset was 428 pg/mL (IQR 2112 to 1465). Like BNP, analysis showed a non-significant average reduction of 15 pg/mL (95% CI −205 to 2 pg/mL, *p* = 0.749). Most subjects (N = 14, 61%) also demonstrated reductions in NT-proBNP levels over time (Figure 1).

### 3.3. Comparison of Baseline Characteristics Between Patients with and Without Follow-Up Data

To evaluate potential bias related to the limited availability of follow-up data, we performed a formal comparison of baseline characteristics between patients with and without follow-up measurements for both 6 min walk distance (6MWD) and BNP. No significant differences were observed between groups in the BNP analyses. For 6MWD, baseline characteristics were compared between 48 patients who had at least one follow-up 6MWD measurement at any time point and 35 patients without follow-up 6MWD data. Baseline demographic and anthropometric characteristics were largely similar between patients with and without complete follow-up, with no meaningful differences in sex, weight, height, body mass index, or body surface area (all SMD < 0.25). There was, however, a trend toward older age among patients in the group with follow-up data (72.8 ± 9.8 vs. 68.6 ± 11.0 years; *p* = 0.071; SMD = 0.40) (Table 3). In contrast, baseline 6MWD was significantly higher in patients with complete follow-up compared with those without follow-up data (250.1 ± 110.0 m vs. 160.9 ± 96.6 m; *p* = 0.016; SMD = 0.86) (Table 3).

### 3.4. Sensitivity Analyses

Among the 48 patients with complete 6MWD data, the median follow-up interval was 234 days (IQR: 175–406 days), with substantial variability (range: 13–1397 days).

The time × follow-up duration interaction was statistically significant (β = −2.3 m/month, SE = 1.12, *p* = 0.043), indicating that the magnitude of change in 6MWD varied depending on when follow-up was assessed, with greater declines observed at longer follow-up intervals.

### 3.5. Tolerance

At the time of the censor, sixty-four patients (77%) remained on therapy, with a mean duration of 533 days (SD 317 days) of inhaled treprostinil exposure, and a collective exposure in this cohort of 85.2 patient-years. Over the course of the study a total of 24 subjects (29%) experienced mortality events (Table 2). The mean duration of inhaled treprostinil exposure for those who electively stopped therapy over the course of the study (N = 19) was 100 days (SD 260 days), and the most reported reason for cessation was cough, followed by chest pain/dyspnea, and medication inconvenience (Table 4).

The majority of this cohort (N = 62, 75%) was able to reach their target dose, defined as nine breaths for iNeb and 48 mcg for dry powder inhaler. Additionally, fifteen patients intolerant to iNeb were transitioned to DPI, and the majority (N = 13, 87%) successfully transitioned, remaining on therapy until the study censor for a mean duration of 433 days (SD 144 d) on DPI.

## 4. Discussion

This retrospective, multicenter study provides real-world data in patients with PH-ILD treated with inhaled treprostinil and highlights that application of prospective randomized clinical trial results in regular clinical practice is inherently challenging. Patients in randomized controlled trials (RCTs) represent a highly selected subset with strict eligibility criteria, preserved function, and fewer comorbidities. Real-world PH-ILD populations are more heterogeneous, with diverse ILD subtypes, variable lung and vascular involvement, and high comorbidity burden. Trial protocols—fixed dosing, intensive monitoring, and frequent follow-up—may not reflect routine practice, so efficacy and tolerability can differ outside controlled settings.

Despite multiple clinical trials in PH-ILD, no other treatment besides inhaled treprostinil has proven effective in an RCT, with some agents showing evidence of harm, including a signal for increased mortality with riociguat and increased hospitalization or clinical deterioration with ambrisentan [1,2,4]. The landmark INCREASE trial [11] enrolled 326 patients with PH-ILD (randomized 1:1 to inhaled treprostinil vs. placebo), with a baseline mean 6MWD of 259.6 m, and mean PVR of 6.2 Wood units. Most common types of ILD were idiopathic interstitial pneumonia (IIP) ~45%, combined pulmonary fibrosis and emphysema (CPFE) ~25%, and connective tissue disease-associated ILD ~22%. The change from baseline to week 16 in peak (i.e., measured after inhalation) 6MWD (the primary endpoint) was 31.12 m (95% confidence interval [CI], 16.85 to 45.39; *p* < 0.001). Patients in the INCREASE trial assigned to inhaled treprostinil, as compared with those in the placebo arms, showed significant improvements in secondary end points, such as a decrease in NT-proBNP, a lower risk of clinical worsening, and demonstrated less exacerbations of the primary lung disease. Based on this trial, the drug was approved for use in PH-ILD. The open label extension (OLE) of the INCREASE trial [14] was designed to capture long-term efficacy and safety data of inhaled treprostinil in PH-ILD; 242 patients who completed the 16-week RCT were enrolled in the OLE and received inhaled treprostinil. Follow-up 6MWD data is available at 52 weeks in the OLE, counted from the beginning of the RCT. The 6MWD for patients originally randomized to active drug showed relative stability in the long-term, with a change of 22.1 ± 66.3 m compared to the baseline in the RCT. In the former placebo arm, open label administration of inhaled treprostinil resulted in a transient improvement in the 6MWD at weeks 28 and 40, which was not sustained at 52 weeks; overall they walked 19.5 ± 69.6 m fewer compared to the RCT baseline. NT-proBNP levels at 64-week follow-up in OLE were stable in the former active arm and decreased appropriately in the former placebo arm. Importantly, in the OLE, patients in the former active arm versus placebo patients in the RCT had a 31% relative risk reduction in exacerbation of the underlying lung disease (HR 0.69, 95% CI = 0.49–0.97, *p* = 0.03), suggesting that a 16-week treatment delay has deleterious effects beyond reduction in the exercise capacity. First, our analyses were notable for low numbers of patients with consistent timeframes of data collection for 6MWD and BNP/NT-ProBNP. After excluding outliers, 25 of 83 patients (30%) had completed 6 min walk testing within six months both before and after inhaled treprostinil initiation, and only seven patients (8%) had assessments obtained within three months of treatment initiation. While 41 of 83, (49%), had available and timely laboratory data, these were further split between BNP and NT-proBNP. Multiple factors contributed to the lack of timely data points including patient visit timing, COVID pandemic restrictions, telemedicine care, comorbidities, as well as infrequent use of 6MWD and BNP assessments in ILD prior to PH-ILD diagnosis. Additionally, during the prolonged process of insurance approval for inhaled treprostinil, there are many steps which must be completed, and while clinically relevant, a 6MWD or NT-proBNP are not required for approval and may have been delayed until next clinical reassessment [15]. Due to lack of consistently timed data, we were unable to make any conclusions on the walk distance given the variability in the data but did report it descriptively. We identified a notable difference in respect to the baseline functional status as measured by 6MWD between patients who had follow-up 6MWD measurement at any point in time (n = 48), versus patients missing follow-up 6MWD determination (n = 35). Patients with complete follow-up had significantly higher baseline 6MWD (250.1 ± 110.0 m vs. 160.9 ± 96.6 m, *p* = 0.016; SMD = 0.86), suggesting that patients with better baseline functional capacity were more likely to complete follow-up testing.

When assessing 6MWD, pharmacokinetics of inhaled treprostinil needs to be considered. It reaches a peak plasma concentration within minutes and drops significantly after one hour. Its trough concentration occurs approximately four hours after treatment [16]. The INCREASE investigators accounted for this in their protocol and required that patients receive treatment within sixty minutes prior to follow-up 6MWDs. In addition to this, they also collected an additional 6MWD at week 15 at trough plasma concentration, four hours after the most recent treatment. While only one week apart, a gap of nearly 10 m was demonstrated between the least mean square differences in the studies, 31 m for the peak walk, and 22 m for the trough walk [11]. Sakao et al. recently demonstrated that improvements in hemodynamics abate as early as 30 min after dosing [17]. In a separate RCT on the use of inhaled treprostinil in pulmonary arterial hypertension, investigators’ protocol also utilized a peak walk test, within 10 to 60 min of treatment [18]. It is unlikely that patients who are using the inhaled nebulizer at home are completing this 6MWD within sixty-minutes of the last treatment and this can have a significant impact on walk distance. In another real-world study evaluating inhaled treprostinil in 42 patients with PH-ILD after 6 months of therapy, efficacy analyses were restricted to 26 patients [19]. This level of attrition mirrors our findings and reflects the methodological constraints and data challenges that commonly arise in routine clinical practice. Notably, their cohort differed from ours in demographic and disease characteristics, with a higher proportion of women (62%) and a greater prevalence of connective tissue disease-associated ILD (55% vs. 24% in our study). Clinical response was defined as an absolute increase in 6MWD of ≥30 m from baseline and was observed in 10 of 26 patients (38.5%). In our cohort, a smaller proportion of patients (7/37, 19%) had an increase in the 6MWD at follow-up in patients with walk testing within one year before and after therapy.

As an additional measure of efficacy, we evaluated changes in levels of natriuretic peptides with therapy. In the INCREASE trial [11], inhaled treprostinil was associated with significant reductions in NT-proBNP during the randomized phase, and these improvements were generally sustained during the open-label extension [14], supporting a treatment effect on right ventricular stress beyond changes in exercise capacity alone.

Due to different institutional policies, in our study, collection of BNP versus NT-proBNP biomarkers was not uniform at all sites, hence we had two different subsets of patients, one with BNP data (n = 25) and another one with NT-proBNP data (n = 23). The majority of our subjects, 63%, had an absolute improvement in their BNP level, over a mean duration of 170 days. This reflects improvement in right ventricular function.

Independent of the functional and laboratory outcomes, we collected data showing that this medication is well-tolerated. In our study, the majority of patients were able to tolerate target doses of inhaled treprostinil for more than a year. Our adherence rate of 77% is nearly identical to what was observed in the INCREASE trial and the open-label extension (OLE) which demonstrated 75.5 and 78% adherence respectively [11,14]. When combining both delivery methods, 74% of our subjects reached a target dose. This is slightly lower than the 81% of patients who were tolerant of 9 breaths in the INCREASE open-label extension but may reflect provider-driven titration patterns that deviate from the INCREASE protocol [11]. Overall, this represents an encouragingly high degree of adherence and tolerance with inhaled treprostinil therapies, regardless of formulations and despite the majority of the cohort experiencing at least one side-effect from therapy. While most were able to tolerate target doses for more than a year, about one-fourth (23%) of patients did elect to discontinue therapy, citing cough, chest pain/dyspnea, and inconvenience as the primary reasons for stopping. This is very similar to the discontinuation rates in the INCREASE trial and INCREASE OLE of 23–24% and 22% respectively [11,14]. Interestingly, in a subset of 15 patients intolerant of iNeb, most (N = 13, 87%) successfully transitioned to DPI and were able to remain on therapy over the course of the study, with a mean duration of roughly 1.5 years. This suggests DPI may be a reasonable alternative to attempt in patients intolerant to iNeb, due to either side-effects or inconvenience, and may enhance medication persistence.

Taken together, these observations suggest that real-world data may not reliably replicate the results seen in randomized controlled trials. Methodological challenges—including incomplete or missing data, heterogeneous patient populations, variable follow-up intervals, and differences in monitoring or treatment adherence—can substantially limit the interpretability of real-world findings. This reality begs the question, “What is a realistic and practical clinical outcome of interest in patients with PH-ILD?”

While our investigation did not yield the same results that were demonstrated in INCREASE, understanding the clinical impact of this new therapy remains paramount for the PH community. However, due to the obstacles that these complex patients face, prospective observational studies with preplanned, peak therapy 6MWD tests, and outpatient laboratory testing with consistent natriuretic peptides are necessary to produce high-quality data to guide clinicians. In this context, several post hoc analyses of the INCREASE trial and the OLE data suggest potential additional benefits, including reductions in clinical worsening events and preservation of forced vital capacity (FVC). In a post hoc analysis of the INCREASE trial [20], inhaled treprostinil was associated with a lower risk of disease progression events, including clinical worsening, hospitalization, lung disease exacerbation, and death. Over the 16-week randomized period, treatment reduced the risk of a first progression event (HR, 0.71; 95%CI, 0.54–0.94; *p* = 0.019), as well as that of recurrent events (HR, 0.53; 95% CI, 0.35–081; *p* = 0.003). These exploratory findings align with the INCREASE OLE [14], in which earlier initiation of inhaled treprostinil was associated with more favorable long-term functional and clinical outcomes, suggesting potential benefits beyond short-term exercise capacity.

In another post hoc analysis of the INCREASE trial, inhaled treprostinil was associated with relative preservation or modest improvement in forced vital capacity (FVC) compared with placebo during the 16-week randomized period, with IPF subgroups showing placebo-corrected differences of ~84.5 mL at week 8 and ~168.5 mL at week 16 in absolute FVC change [21]. In the INCREASE open-label extension [14], mean absolute FVC change from randomized baseline to week 64 was approximately +51 mL, corresponding to a FVC% mean increase of 2.8 ± 8.6%, with more favorable trajectories observed among patients who initiated therapy earlier rather than after a delayed start. These collective findings, together with preclinical evidence of antifibrotic effects, have informed the design of the TETON phase 3 platform [22], in which the primary endpoint is change in absolute FVC at week 52 in patients with idiopathic pulmonary fibrosis (IPF) or progressive pulmonary fibrosis (PPF).

In this retrospective, multicenter, real-world study, we observed that inhaled treprostinil was well-tolerated in patients with PH-ILD; the majority of patients were able to reach target dose and remain on medication for more than a year regardless of delivery method and despite the majority endorsing at least one adverse side-effect of therapy. Encouragingly, many of those intolerant to iNeb were able to successfully transition to DPI. Despite this partial tolerance, there was a trend for improvement in brain natriuretic peptides. The 6MWD data interpretation was limited by heterogeneity of timing of data which was likely impacted significantly by COVID pandemic face to face visit and testing limitations.

Although our study has several strengths, including a large cohort of patients with real-world data and a lengthy duration of follow-up, a number of limitations should be considered. Due to variability in adequate timing between baseline and follow-up testing, our analyses of changes in 6MWD and natriuretic peptides were limited by small numbers and should be interpreted with caution. Similarly, small numbers prevented direct comparison between iNeb and DPI formulations of treprostinil.

In summary, although we could not replicate the improvement in the 6 min walk test noted in the INCREASE trial, there was a modest signal in right ventricular biomarker response. Methodological limitations inherent to retrospective analyses—such as non-standardized assessments and missing data—as well as patient-level factors including higher comorbidity burden, more advanced disease, and survivor bias, may attenuate detectable treatment effects despite a clinically meaningful impact. Additionally, there is evidence that, in PH-ILD, inhaled treprostinil may provide benefits beyond short-term exercise capacity, such as reduction in the disease progression, lower risk of exacerbation of the underlying lung disease, or potentially an antifibrotic effect. It is also important to recognize that 6MWD may have limitations as a measure of therapeutic response in this patient population. In patients with PH-ILD, functional capacity and distance walked are influenced not only by PH, but also by underlying ILD, musculoskeletal factors, and comorbidities. As a result, changes in 6MWD may underestimate—or fail to fully capture—the clinical benefits of inhaled treprostinil, highlighting the need for complementary endpoints to assess efficacy more comprehensively in this complex population. In clinical practice, all these factors would have to be considered and balanced against cumbersome administration and significant cost [23]. Given the complexity of individual patients with PH-ILD, the highly variable therapeutic response, and the limited treatment options, optimal management of these patients should, ideally, involve multidisciplinary meetings and delineation of clinical protocols for evaluation and prescribing inhaled treprostinil.

## 5. Conclusions

To our knowledge, this is one of the very few studies reporting the real-world outcomes of inhaled Treprostinil in PH-ILD patients. Over long periods of time, 6MWD may not be the optimal marker of treatment response in PH-ILD patients on inhaled treprostinil therapy. We observed that inhaled treprostinil is well-tolerated, with a high percentage of patients remaining on therapy and reaching target dose. The DPI formulation appears to be a reasonable alternative for those not tolerating nebulized therapy. Further work will be needed to determine the optimal strategy to monitor treatment effectiveness and the preferred metric for treatment response (e.g., hospitalizations, quality of life, biomarkers) of inhaled treprostinil in the PH-ILD population.

## Figures and Tables

**Table 1:** Baseline demographics and disease characteristics.

Baseline Characteristics	All Patients (N = 83)
Age—median (IQR)	73 (10)
Sex—no. (%)	
Female	38 (45.8)
Male	45 (54.2)
Race/Ethnicity—no. (%)	
White	61 (73.5)
Black or African American	12 (14.5)
Hispanic or Latino	5 (6)
Asian	5 (6)
Etiology of lung disease—no. (%)	
Idiopathic pulmonary fibrosis	28 (33.7)
Connective tissue disease	20 (24.1)
Chronic hypersensitivity pneumonitis	8 (9.6)
Combined pulmonary fibrosis and emphysema	12 (14.5)
Nonspecific interstitial pneumonia	5 (6)
Supplemental oxygen use—no. (%)	77 (92.8)
Baseline medical therapy—no. (%)	
Antifibrotics	28 (34)
Pirfenidone	9 (11)
Nintedanib	19 (23)
Immunosuppression	29 (35)
Baseline Disease Severity—mean (SD)	
FVC % predicted (L)	65 (14)
DLCO % Predicted (mL/min/mmHg)	34 (14)
Mean pulmonary artery pressures (mmHg)	34 (8.4)
Pulmonary Vascular Resistance (WU)	5.3 (2.5)
Cardiac index (L/min/m^2^)	2.5 (0.7)
Right ventricular systolic pressure (mmHg)	60 (20.5)

**Table 2:** Medication details.

Medication Details	All Patients (N = 83)
Delivery method—no. (%)	
Inhaled nebulizer (iNeb)	75 (90)
Dry powder inhaler (DPI)	8 (10)
Transitioned from iNeb to DPI	15 (18)
Medication status—no. (%)	
Still on therapy	64 (77)
Stopped therapy	19 (23)
Still on therapy after transitioning iNeb to DPI	13 (87)
Patient status—no. (%)	
Alive	59 (71)
Deceased	24 (29)
Duration of therapy—median (IQR)	
Stopped therapy (days)	100 (40–156)
Still on therapy (days)	533 (257–740)
Maximum dosage tolerated	
Inhaled nebulizer—no. (%)	
3–6 breaths	11 (17)
7–9 breaths	21 (32)
10–12 breaths	23 (35)
≥13 breaths	10 (15)
Dry powder inhaler—no. (%)	
≤32 mcg	2 (11)
48 mcg	1 (6)
64 mcg	10 (56)
≥80 mcg	5 (28)

**Table 3:** Comparison of baseline characteristics between patients with and without complete 6 min walk distance (6MWD) follow-up data.

Variable	Without Follow-Up (n = 35)	With Follow-Up (n = 48)	*p*-Value	SMD
Age, years	68.6 ± 11.0	72.8 ± 9.8	0.071	0.40
Female sex, n (%)	16 (45.7%)	22 (45.8%)	1.000	0.00
Weight, kg	78.2 ± 22.2	78.0 ± 17.3	0.949	0.01
Height, cm	170.1 ± 9.8	167.7 ± 10.2	0.299	0.23
BMI, kg/m^2^	26.8 ± 6.3	27.7 ± 5.8	0.486	0.16
BSA, m^2^	1.89 ± 0.29	1.86 ± 0.23	0.684	0.09
Baseline 6MWD, m	160.9 ± 96.6	250.1 ± 110.0	**0.016**	**0.86**

Continuous variables presented as mean ± SD. SMD = standardized mean difference. Bold indicates *p* < 0.05 or SMD > 0.20.

**Table 4:** Reported adverse effects.

Adverse Events—No. (%)	All Patients (N = 83)	Inhaled Nebulizer (N = 75)	Dry Powder Inhaler (N = 8)
Cardiopulmonary Hospitalization	41 (49)	38 (51)	3 (38)
Chest Pain	7 (8.4)	6 (8)	1 (13)
Dyspnea	16 (19)	16 (21)	0
Hypotension	5 (6)	5 (7)	0
Nausea	16 (19)	15 (20)	1 (13)
Dizziness	19 (23)	19 (25)	0
Cough	51 (61)	44 (59)	7 (88)
Headache	19 (23)	17 (23)	2 (25)
Throat irritation	24 (29)	23 (31)	1 (13)
Fatigue	9 (11)	9 (12)	0
Diarrhea	16 (19)	15 (20)	1 (13)
Patients who stopped therapy	N = 19	N = 17	N = 2
Reasons for stopping—no. (%)			
Cough	11 (58)	10 (59)	1 (50)
Chest pain/dyspnea	7 (37)	7 (39)	0
Inconvenience	5 (26)	4 (22)	1 (50)
Throat Irritation	2 (11)	2 (11)	0
Nausea	2 (11)	2 (11)	0
Dizziness	1 (5)	1 (6)	0
Hypotension	1 (5)	1 (6)	0
Headache	1 (5)	1 (6)	0
Other			
Perioral rash	1 (5)		
Anxiety	1 (5)		
Worsening hypoxia	1 (5)		

## Data Availability

For interest in raw data gathered for this article, please reach out to the senior author, Namita Sood, nsood@health.ucdavis.edu.

## Abbreviations

The following abbreviations are used in this manuscript:

6MWD	Six minute walk distance
BNP	B-type natriuretic peptide
CI	Cardiac index
DPI	Dry powder inhaler
D	Days
ILD	Interstitial lung disease
iNeb	Inhaled Nebulizer
IQR	Interquartile range
IRB	Institutional Review Board
NT-proBNP	N-terminal natriuretic peptide
OLE	Open-label extension
PAH	Pulmonary arterial hypertension
Pg	Picograms
RCT	Randomized control trial
SD	Standard deviation
SMD	Standardized mean differences
